# Consensus Description of Inclusion and Exclusion Criteria for Clinical Studies of Nonallergic Rhinopathy (NAR), Previously Referred to as Vasomotor Rhinitis (VMR), Nonallergic Rhinitis, and/or Idiopathic Rhinitis

**DOI:** 10.1097/WOX.0b013e3181b2ff8a

**Published:** 2009-08-15

**Authors:** Michael A Kaliner, James N Baraniuk, Michael S Benninger, Jonathan A Bernstein, Phil Lieberman, Eli O Meltzer, Robert M Naclerio, Russell A Settipane, Judith R Farrar

**Affiliations:** 1 Institute for Asthma and Allergy, 5454 Wisconsin Ave, Suite 1700, Chevy Chase, MD, Washington, DC; 2 Division of Rheumatology, Immunology and Allergy, Georgetown University, Washington, DC; 3 Head and Neck Institute, The Cleveland Clinic, Cleveland, OH; 4 University of Cincinnati College of Medicine, Cincinnati, Ohio; 5 Division of Allergy and Immunology, University of Tennessee, Germantown, TN; 6 Allergy & Asthma Medical Group & Research Center, San Diego, CA; 7 Section of Otolaryngology--Head and Neck Surgery, University of Chicago, Chicago, IL; 8 Brown Medical School Allergy & Asthma Center, Providence, RI, Washington, DC; 9 LifeSciences Press, Washington, DC

**Keywords:** nonallergic vasomotor rhinitis, nonallergic rhinitis, vasomotor rhinitis, idiopathic rhinitis, nonallergic rhinopathy

## Abstract

"Nonallergic rhinopathy" was defined by consensus at a Roundtable conference in December 2008 as "a chronic nasal condition with symptoms that may be perennial, persistent, intermittent or seasonal and/or elicited by recognized triggers." The definition includes a well-recognized set of clinical exposures that lead to the symptoms, predominantly congestion, rhinorrhea, and postnasal drip. These clinical characteristics help to identify patients for participation in clinical trials examining the efficacy of treatments for this important disease. The next step is to establish inclusion and exclusion criteria that will provide a framework for the clinical trials. Agreement on study criteria was obtained at the consensus conference by discussion, counterpoint, and compromise.

## Introduction

To advance knowledge of a clinical entity, we must begin with a definition. From this definition, we next design clinical trials to study pathophysiology and/or examine therapeutic options. The results of clinical trials help us to better understand the disease, and might lead to refining the definition and the study parameters, notably the inclusion/exclusion criteria.

As noted in previous articles in these Proceedings, the medical literature contains various contradictory opinions regarding the definition of vasomotor rhinitis (VMR) and its pathophysiology, a fact that has complicated determination of inclusion and exclusion criteria for clinical trials. In general, expert opinion, guidelines, and published studies have sup-ported a definition in which VMR subjects are described as experiencing worsening rhinitis symptoms related to both weather/temperature triggers (eg, changes in temperature, barometric pressure, and humidity) and odor/irritant triggers (eg, strong perfume, hot/spicy food, alcoholic beverages, smoke, and other airborne irritants) [1-9]. The same clinical description "by triggers" has been applied to nonallergic rhinitis (NAR). There is only a clinical impression that these triggers represent different disease processes, unsupported by any pathophysiologic investigations.

Employing a definition based upon triggers alone may be acceptable from a clinical practice standpoint, but it is problematic from the perspective of developing appropriate clinical trials to evaluate new treatment options. First, the pathways by which the triggers cause nasal symptoms are not clearly understood and may or may not differ. Second, there are many stimuli which cause the spectrum of symptoms clinically recognized as VMR, as illustrated in Table 1, which shows the variety of opinions reported in the published literature regarding factors that can result in a nasal vasomotor response. Despite differences in the authors' classifications of the response as NAR or VMR, it is evident that the authors are describing the same symptomatic phenomena. In most cases, the choice of the specific term (VMR or NAR) seems arbitrary; and although VMR is often used in relationship to weather or temperature change, the resulting symptoms overlap with those caused by other nonallergic stimuli.

**Table 1:** Triggers Included in Articles Describing Nonallergic (Vasomotor) Rhinitis

		Type of Triggers Included							
		
Paper	Description o:p	Pollution	Chemical	Olfactory	Temperature	Weather	Work-Related	Food	ETS
Bachert, 2004 [7]	Review	-	+	-	+	+	+	-	-
Banov et al, 2001 [10]	Prospective study*	-	+	-	+	+	-	+	-
Bonini et al, 2006 [11]	Athletes	-	+**	-	+	-	+**	-	-
Brandt and Bernstein, 2006 [1]	Prospective study*	+	+	+	+	-	-	+	+
Bousquet et al, 2008 [6]	Review	+	+	+	+	-	-	-	+
Ciprandi, 2004 [12]	Review	-	+	+	+	+	-	+	-
Garay, 2004 [13]	Review	-	+	-	+	-	+	-	+
Greiner and Meltzer, 2006 [14]	Review	-	-	+	-	+	-	+	+
Kaliner, 2007 [8]	Review	-	+	+	+	+	-	+	-
Molgaard et al, 2007 [15]	Prospective study*	-	-	-	-	-	-	-	-
Newhall and McGrath, 2004 [9]	Review	+	+	+	-	+	-	+	+
Rondon et al, 2007 [16]	Prospective study*	-	-	-	+	-	-	+	-
Settipane, 2001 [17]	Review	-	+	-	+	-	-	+	-
Webb et al, 2002 [18]	Analysis of 3 pooled prospective studies*	-	-	-	-	-	-	-	+
Total for triggers		3	10	6	8	6	2	6	5

*Prospective trial in which nonallergic rhinitis was defined as symptoms from irritant triggers with negative allergy tests.

**Study of athletes: exercise-induced symptoms are listed as work-related in this table, and chemicals were swimming pool-related OK?

As noted in the Consensus Definition from these Proceedings published in the June issue of the *World Allergy Organization *journal the lack of straightforward diagnostic criteria is limiting; research for better treatment options requires the definition of "homogeneous" populations characterized by well-defined inclusion and exclusion criteria. Based on expert opinion and review of the published literature, the attendees of the Roundtable proposed to revise the terminology to reference this condition as "nonallergic rhinopathy," which they defined as "a chronic nasal condition with symptoms that may be perennial, persistent, intermittent or seasonal and/or elicited by recognized triggers." According to this definition, the symptoms of nonallergic rhinopathy, predominantly congestion, rhinorrhea, and postnasal drip, may occur in response to a well-recognized set of clinical exposures, may be associated with several comorbidities, and can be distinguished from other well-defined clinical causes of rhinopathy (see Part 1 of these Proceedings in the June issue of this journal for greater detail). Those clinical characteristics, as outlined in Table 2, can serve as the basis for well-defined inclusion and exclusion criteria that should permit precise identification of patients for participation in clinical trials. As experience is gained from trials, these initial starting criteria may need to be altered.

**Table 2:** Clinical Characteristics of Nonallergic Rhinopathy (Referred to in This Table as NAR)

Symptoms of NAR may be perennial, persistent, seasonal (i.e., seasonal climatic changes-see below), intermittent and/or elicited by defined triggers that may include:
• Cold air
• Changes in climate (temperature, humidity, barometric pressure)
• Strong smells (such as perfume, cooking smells, flowers, chemical odors)
• Environmental tobacco smoke
• Changes in sexual hormones levels
• Pollutants and chemicals (e.g. volatile organics)
• Exercise
• Alcohol ingestion
NAR may present with concomitant conditions such as:
• Food-related rhinorrhea
• Mild nasal eosinophilia (< 5%)
• Eustachian tube dysfunction (ear pressure/popping/pain)
• Senile rhinitis
NAR symptoms are not caused by other known etiological causes for rhinopathy, such as:
• Chronic rhinosinusitis or nasal polyps
• NARES (nasal eosinophilia > 5%)
• Aspirin-related chronic rhinosinusitis, nasal polyps, or asthma (although NAR is often seen as one of the clinical characteristics of AERD)
• Infectious rhinitis or rhinosinusitis (e.g., viral upper respiratory infections, bacterial/fungal rhinosinusitis, bacterial rhinitis)
• Anatomical abnormalities
• Drug usage, (e.g., adverse effect of systemic medication, excess use of topical decongestants)
• Cerebrospinal fluid leak
• Pregnancy

## Discussion and Consensus

Once the definition of nonallergic rhinopathy was agreed upon, discussion at the Roundtable Meeting focused on specific inclusion and exclusion criteria for study protocols. Initially, it was suggested that to create a study to evaluate the causes of, or potential treatment for, nonallergic rhinopathy, each individual precipitating factor might need to be investigated independently. In other words, the inclusion and exclusion criteria for each study would reflect the specific trigger of symptoms, and the trial endpoints would measure the patient's response to that particular trigger. Based on this viewpoint, the duration of each study also would depend upon the specific trigger. Weather/temperature triggers tend to be more persistent than odor/irritant triggers; therefore, studies involving the former would be of longer duration than the latter. An example of a clinical study using weather-related changes as the trigger was presented at the meeting to illustrate these points.

Discussion, occasionally heated, followed this suggested approach to the study design. Some participants disagreed with the idea that 2 or more study designs are needed for clinical studies of nonallergic rhinopathy; one for weather and related triggers and another for the transient types of triggers. This group stated that the range of triggers should be included as entry criteria and not as endpoints, the endpoints being changes in nasal symptoms and quality of life. In this regard, the disease state (nonallergic rhinopathy) would be treated like asthma for developing studies of new medications to treat the clinical disease. Patients who are nonallergic and who have appropriate chronic nasal symptoms, with no evidence of mechanical obstruction or infectious disease, would be studied using standardized, controlled protocols, regardless of the trigger. If their symptoms improve with statistically significant differences from placebo, then the outcome is considered positive, and additional post hoc analyses/stratification (eg, based on specific triggers, blood eosinophils, nasal secretions, and other possible stratifying criteria) could also be performed. Other discussants emphasized that not every clinical study of nonallergic rhinopathy study could be performed in exactly the same manner. This group agreed that simplifying the study design was needed but remained concerned that including multiple triggers would introduce a type 1 error. Furthermore, the group was nearly unanimous in pointing out that nonallergic patients who have no recognized triggers represent a legitimate portion of the nonallergic rhinopathy population.

It was eventually agreed that the objective of this consensus paper is how to design a study that is inclusive of all patients with nonallergic rhinopathy to develop new therapies. In this regard, then, it is not necessary to stratify patients based on each specific trigger. Consensus was achieved on the following.

• In terms of efficacy protocols, triggers represent an inclusion criteria and not a specific outcome. However, triggers could be evaluated by post hoc subgroup analysis, as appropriate.

• The endpoints of these studies are symptom score dependent and should include the total nasal symptom score, the individual nasal symptoms, and a quality of life instrument such as the Rhinosinusitis Disability Index, The Sino-nasal Outcomes Test, or the Rhinoconjunctivitis Quality of Life Questionnaire.

• Challenge studies with specific triggers such as might be examined in chamber studies are not included in this discussion and represent a separate entity in which the trigger-induced symptoms might be evaluated as primary endpoints.

• The inclusion criteria should be based on the characteristics identified in the consensus definition of nonallergic rhinopathy as shown in Table 2.

• Post hoc evaluation may be appropriate to assess individual triggers and/or concomitant conditions (eg, gusatory rhinitis).

• Patients with chronic nonallergic rhinopathy without triggers would not be included in these initial studies, but the group recognized that nonallergic rhinopathy without triggers is a part of the spectrum of nonallergic rhinopathy and that it would be appropriate to develop other clinical protocols in which the lack of triggers is part of the inclusion/exclusion criteria.

• The group agreed that nonallergic rhinopathy with nasal eosinophilia (NARES) may well be a different pathophysiologic entity. Until the relationship between nonallergic rhinopathy and NARES are clearly established, the group felt that nasal eosinophilia would be an exclusion criteria, and those patients with NARES should be studied in separate protocols.

In summary, the essential characteristics for inclusion in clinical trials of nonallergic rhinopathy are: 1) no allergic rhinitis; 2) negative prick skin tests or serum antigen specific IgE assays; and 3) a series of symptoms, in a patient with nonmechanical, noninfectious rhinitis.

Table 3 presents the entry criteria for an efficacy study protocol for nonallergic rhinopathy based on the above consensus points. For the purpose of listing inclusion and exclusion criteria, it is assumed that the clinical study will be a typical blinded, repeat dosing, parallel group or crossover, outpatient study. The study should include a run-in period long enough to establish that the subjects met a specific level of nasal symptomatology (1-4 weeks) followed by a 2-4-week treatment period. Exacerbations of the patient's symptoms would be collected in the daily diary, and specific triggers associated with the exacerbation would be recorded before the study and during the study for possible post hoc evaluation of specific triggers. However, in counterpoint, it was noted that many of these patients already try to avoid their triggers as much as they can, so it was not clear that collecting data on exacerbations would be helpful. Significant error could result from problems with poor recall and negative memory of an aversive stimulus. As such, the use of objective instruments for quantifying the degree of symptoms is preferred.

**Table 3:** Proposed Consensus Criteria for an Efficacy Study of Therapy for Nonallergic Rhinopathy (Referred to in This Table as NAR)

Inclusion: Subjects should meet all criteria listed below:
1. Diagnosis of non-allergic rhinopathy (NAR) as defined to include all of the following:
a. Two year clinical history of NAR symptoms, including nasal congestion, nasal discharge, and post-nasal drip
b. Chronic, perennial nature of symptoms with fluctuation/exacerbation related to one or more triggers including: cold air, changes in climate (temperature, humidity, barometric pressure), strong smells (such as perfume, cooking smells, flowers, chemical odors), exposure to environmental tobacco smoke, changes in sexual hormones levels, exposure to pollutants and chemicals (e.g. volatile organics), abnormal nasal response to exercise or alcohol ingestion.
c. Negative skin prick tests to seasonal allergens (e.g., trees, grass, weed, etc) and perennial allergens (e.g., animal dander, house dust mite, cockroach, etc). Negative test defined as a wheal < 3 mm larger than the diluent control or negative serum specific IgE antibody levels.
d. Positive response to histamine skin prick test. Positive test defined as wheal ≥ 3 mm larger than the diluent control.
e. Normal sinus radiograph (Waters view or CT) to rule out sinusitis
2. Nasal cytology negative for eosinophils to rule out NARES (less than 5% of total cells)
3. Informed consent: Appropriately signed and dated informed consent for study subjects ages 18 years and older. For study subjects ages less than 18 years, informed consent signed by parents or care providers
4. Subjects are able, willing, and likely to comply with study procedures and restrictions
5. Subjects can be treated on outpatient basis
6. Age 12 years or older
7. Female of childbearing potential must commit to using acceptable method of birth control [method may vary depending on marketing status and teratogenicity potential]
8. Subjects literate enough to read, understand, and record information in native language (or language that will be used in the study procedure)
Exclusion: Subjects will not be eligible for inclusion if any of the following criteria is met:
1. Significant concomitant medical condition defined as but not limited to:
a. History or current evidence of clinically significant disease of any body system that in the opinion of the investigator would put the safety of the study subjects at risk through participation in the study, or would confound the interpretation of the study results.
b. Significant anatomical nasal disease such as physical obstruction of the nose, substantial deviated nasal septum, or nasal septal perforation that could affect the deposition of intranasal study drug and interfere with interpretation of medication outcomes. Rhinitis medicamentosa
c. Common cold or any bacterial or viral infection of the upper respiratory tract for 14 days prior to screening period
d. Documented acute or chronic sinusitis, as determined by Waters view or CT scan
e. Physical impairment that would affect subject's participation in the study
f. History of psychiatric disease, intellectual deficiency, poor motivation, substance abuse, alcohol abuse, cocaine use, or other conditions that would limit the validity of the informed consent, or confound the interpretation of the study
g. Use of intranasal, inhaled, oral, intravenous, intramuscular, ocular, or dermatologic corticosteroid for 30 days prior to screening period
2. Use of other drugs for allergic diseases in the timeframe of the screening period
a. Intranasal or ocular cromolyn within 14 days
b. OTC or prescription antihistamines by any route, including topical within 14 days
c. Oral or nasal decongestants within 3 days
d. Oral, nasal, or inhaled anticholinergics within 3 days
e. Antileukotrienes within 3 days
f. Any immunosuppressants for 6 months
g. Cough and cold lozenges or throat sprays within 3 days
3. Patients likely to use any drugs listed in items 2 above during treatment period
4. Use of drugs likely to affect NAR or its symptoms, such as, but not limited to, tricyclic antidepressants, long-acting beta-agonists, any intranasally administered medication
5. Chronic use of drugs that can cause rhinitis or rhinitis type symptoms, such as, but not limited to, ACE inhibitors, reserpine, guanethidine, methyldopa, hydralazine, beta-blockers, alpha-adreneceptor antagonists, phentolamine, chlorpromazine, aspirin, NSAIDs
6. Use of botanical agents or dietary supplements
7. Allergen immunotherapy
8. Breastfeeding females
9. Positive or inconclusive pregnancy test for females
10. Any affiliation with study investigator or investigational site
11. Tobacco use within one year of the study participation or a total of 10 pack years of use in the past.
12. Use of any investigational or experimental medicine within 30 days
13. Clinically significant abnormality in any clinical laboratory parameter, ECG, urinalysis, or physical examination finding
14. Drug specific exclusion criteria, [for example, for nasal corticosteroids, these would include candida infection of the nose or upper airway, ocular herpes simpex, glaucoma, cataract, shingles, chicken pox, measles, adrenal insufficiency, etc.]

## Conclusions

The Consensus group appreciated the importance of nonallergic rhinopathy as a clinical disease in the US and worldwide [6,17]. Currently, there are no phase 3 trials in the US examining treatment of this disease despite the interest on the part of the pharmaceutical industry and the need by patients for additional treatment options. The Consensus conference was developed with the expectations that 1) an acceptable definition of the disease could be established; 2) creation of appropriate exclusion/inclusion criteria could be based on the new definition; and 3) straightforward criteria will then lead to new interest and the development of more studies in this area. We believe that the criteria published in this paper, taken in the context of the accompanying papers, will facilitate this development and lead to the availability of new clinical choices for treatment of this disease. We also believe - that the process of consensus development can be useful for other entities where clarity is needed.
